# 基于生物信息学分析探索*PCDHGB4*在肺鳞癌发生中的作用

**DOI:** 10.3779/j.issn.1009-3419.2024.102.03

**Published:** 2024-03-20

**Authors:** Ruijiao LU, Xieyidai ABUDUHAILILI, Yuxia LI, Jie NING, Yangchun FENG

**Affiliations:** 830011 乌鲁木齐，新疆医科大学第三临床医学院（附属肿瘤医院）医学检验中心; Medical Laboratory Center, The Third Clinical Medical College (Affiliated Cancer Hospital) of Xinjiang Medical University, Urumqi 830011, China

**Keywords:** PCDHGB4, 肺肿瘤, 预后, PCDHGB4, Lung neoplasms, Prognosis

## Abstract

**背景与目的:**

肺鳞状细胞癌（lung squamous cell carcinoma, LUSC）是非小细胞肺癌（non-small cell lung cancer, NSCLC）的亚型之一。有报道原钙黏蛋白γ家族的成员能通过抑制Wnt信号通路来调节肿瘤细胞的生长，原钙黏蛋白γB4（protocadherin-gamma subfamily B4, PCDHGB4）作为家族成员在LUSC中的研究少有报道，本研究旨在通过生物信息学方法探究PCDHGB4在LUSC发生发展中的作用及潜在的预后价值。

**方法:**

应用癌症基因组图谱（The Cancer Genome Atlas, TCGA）、cBioPortal和UALCAN等数据库，对PCDHGB4在LUSC中的表达与预后、临床病理特征、免疫细胞浸润、免疫调节基因、免疫检查点抑制剂（immune checkpoint inhibitors, ICIs）和甲基转移酶等进行分析。单细胞水平的研究对细胞亚型的聚类结果和PCDHGB4在不同免疫细胞亚群中的表达情况进行了分析。此外，我们还比较了LUSC组织与正常组织中PCDHGB4的启动子甲基化水平，并对其进行了蛋白质-蛋白质相互作用和突变分析。最后基于差异表达基因进行富集分析。

**结果:**

生信分析结果显示PCDHGB4在LUSC组织的表达水平低于正常组织。生存分析显示，PCDHGB4表达增加与患者较差的预后有关。单细胞分析显示，PCDHGB4主要在T细胞、单核细胞或巨噬细胞以及树突状细胞中表达，进一步发现PCDHGB4在肿瘤免疫中发挥着不可忽视的作用，并证实了PCDHGB4与免疫检查点途径基因、免疫调节基因和甲基转移酶有一定的相关性。此外，通过富集分析发现PCDHGB4参与了癌症相关的多条通路。

**结论:**

PCDHGB4在LUSC中低表达，PCDHGB4与患者预后不良有关，并且PCDHGB4与肿瘤免疫细胞浸润和通路密切相关。PCDHGB4可能是LUSC潜在的预后标志物和免疫治疗新靶点。

## Abstract (en)

**Background and objective:**

Lung squamous cell carcinoma (LUSC) is a subtypes of non-small cell lung cancer (NSCLC). It has been reported that members of the protocadherin γ family can regulate tumor cell growth by inhibiting the Wnt signaling pathway. Protocadherin-gamma subfamily B4 (PCDHGB4) as a family member in LUSC was rarely reported. The aim of this study was to investigate the role and potential prognostic value of PCDHGB4 in the development of LUSC using bioinformatics methods.

**Methods:**

The Cancer Genome Atlas (TCGA), cBioPortal and UALCAN databases were used to analyze the expression, prognosis, clinicopathological features, immune cell infiltration, immune regulatory genes, immune checkpoint inhibitors (ICIs), and methyltransferases of PCDHGB4 in LUSC. At the single cell level, we analyzed the clustering results of cell subtypes and the expression of PCDHGB4 in different immune cell subpopulations. In addition, we compared the promoter methylation levels of PCDHGB4 in LUSC tissues and normal tissues and performed protein-protein interaction and mutation analysis. Finally, enrichment analysis was performed based on the differentially expressed genes.

**Results:**

Bioinformatics analysis results showed that the expression level of PCDHGB4 in LUSC tissues was lower than that in normal tissues. Survival analysis showed that increased PCDHGB4 expression was associated with poor prognosis. Single-cell sequencing analysis showed that PCDHGB4 was expressed in T cells, monocytes or macrophages, and dendritic cells. It was further found that PCDHGB4 played an important role in tumor immunity and confirmed that PCDHGB4 was associated with immune checkpoints, immune regulatory genes, and methyltransferases. Besides, enrichment analysis revealed that PCDHGB4 was involved in multiple cancer-related pathways.

**Conclusion:**

The expression of PCDHGB4 was low in LUSC. PCDHGB4 was related to the poor prognosis of patients, and PCDHGB4 was closely related to the infiltration and pathway of tumor immune cells. PCDHGB4 may be a potential prognostic marker and a new target for immunotherapy in LUSC.

肺鳞状细胞癌（lung squamous cell carcinoma, LUSC）是肺癌重要的类型之一，约占非小细胞肺癌（non-small cell lung cancer, NSCLC）患者的30%^[1]^。2022年全球癌症统计数据^[2]^指出，肺癌是最常见的癌症类型之一，也是癌症死亡的主要原因，患者5年生存率低于15%。研究^[3]^表明，肺癌及其主要组织学亚型是携带体细胞突变数量最多的癌症之一，由于高度的基因组复杂性和增强的整体突变负荷，再加上受到吸烟、环境污染和遗传等因素的影响，极易导致疾病向复杂的方向发展，所以以往的治疗方法已经慢慢向靶向治疗与化疗结合的免疫疗法方向发展^[4]^，因此找寻新的免疫治疗靶点显得尤为重要。PCDHGB4是原钙黏蛋白γ基因簇的成员，属于簇状原钙黏蛋白，由923个氨基酸组成，是5号染色体上串联连接的3个相关簇之一，又被称为CDH20或FIB2。其功能主要是参与大脑中特定神经元连接的建立和维持，同时也是潜在的钙依赖性细胞黏附蛋白^[5]^，与生长不良、骨骼异常的神经发育障碍以及乳头状颅咽管瘤等疾病有关。有研究^[6]^发现体内单个原钙黏蛋白γ基因簇亚型过表达可影响大脑皮层中典型Wnt信号通路的因子，而Wnt信号通路的异常激活广泛参与多种肿瘤细胞的增殖、侵袭及耐药等生物学行为，并在肿瘤微环境中起重要调控作用。CDH20/PCDHGB4在宫颈癌样本中表达下调，与宫颈癌临床特征相关，是一种肿瘤抑制因子。与β-连环蛋白相互作用，通过转化生长因子-β/Smad/Snail介导的上皮-间充质转化抑制宫颈癌细胞的迁移和侵袭，推测其在治疗方面存在一定的潜力。其突变也在多种癌症中被报道，包括食管腺癌、结直肠癌、宫颈癌和乳腺癌，其中在41%的食管腺癌中检测到PCDHGB4的拷贝数缺失，并且观察到杂合性缺失，证实了其在食管腺癌进展中的作用^[7,8]^。同时有关PCDHGB4中DNA甲基化的作用在神经发育的差异性中被提到^[9]^。然而，少有研究报道关于PCDHGB4与LUSC的关系以及PCDHGB4在LUSC发生发展中的确切作用。本研究通过生物信息学方法分析PCDHGB4在LUSC发生发展中的作用及预后意义，以期为LUSC患者的预后评估和免疫治疗提供新思路。

## 1 资料和方法

### 1.1 PCDHGB4表达及定位分析

下载33个肿瘤的癌症基因组图谱（The Cancer Genome Atlas, TCGA）RNA-seq数据，同时下载包括49个正常组织和502个LUSC组织的TCGA-LUSC数据集，通过Wilcoxon秩和检验分析PCDHGB4在泛癌和LUSC中的表达情况。使用基因表达综合数据库的GSE10072数据集验证PCDHGB4在LUSC中的表达情况。同时选取GSE33532数据集，该表达谱包括80个NSCLC组织样本和20个匹配的正常肺组织样本，以探讨PCDHGB4在NSCLC中的表达情况。接着使用人类蛋白质图谱数据库研究了PCDHGB4在LUSC中的蛋白表达水平，并通过间接免疫荧光显微镜确定PCDHGB4的亚细胞定位。

### 1.2 PCDHGB4与临床病理特征的关系

从TCGA数据库获取LUSC的临床数据，根据PCDHGB4在所有样本中的中位数将样本分为两组，用卡方检验对PCDHGB4的表达与临床-患者的病理参数关系进行研究，箱型图从UALCAN数据库下载。

### 1.3 PCDHGB4的预后分析

从TCGA数据库中下载了TCGA-LUSC数据集的临床信息，选择总体生存期（overall survival, OS）、疾病特异性生存期（disease specific survival, DSS）和无进展生存期（progression-free survival, PFS）来分析PCDHGB4表达对LUSC患者预后的影响。GEPIA数据库选取肺腺癌和LUSC两个数据集，以探究PCDHGB4的表达与NSCLC患者OS和无病生存期（disease free survival, DFS）之间的关系。此处统计学方法选择Log-rank检验。

### 1.4 PCDHGB4的单细胞分析

使用scTIME提供的GSE127465数据集，分析细胞亚型的聚类结果和PCDHGB4在不同免疫细胞亚群中的表达情况。接着使用肿瘤微环境单细胞数据库分析PCDHGB4在每种细胞类型中的表达水平，通过热图进行定量和可视化。

### 1.5 PCDHGB4的表达与免疫的关系

利用R“GSVA”包通过单样本基因集富集并应用Spearman方法探索PCDHGB4与24种免疫细胞浸润之间的关系，Wilcoxon秩和检验比较LUSC中高和低PCDHGB4表达组之间免疫细胞浸润的差别。接着利用ESTIMATE算法推断LUSC样本的免疫浸润得分，包括基质得分、免疫浸润得分和估计得分。此外，从加州大学圣克鲁兹校区下载了经统一标准化的LUSC数据集，将表达值进行了log2（x+0.001）变换后采用Spearman法研究PCDHGB4与免疫调节基因之间的关系，包括主要组织相容性复合物、免疫刺激剂、免疫抑制剂、趋化因子和趋化因子受体。

### 1.6 基于免疫检查点基因表达预测肿瘤免疫治疗价值

Sangerbox数据库获取ENSG00000253953（PCDHGB4）基因和60个两类免疫检查点途径基因[抑制性（24）、刺激性（36）]的表达数据，将表达值进行了log2（x+0.001）变换并计算Spearman相关性。接着使用TIMER和TCGA数据库对LUSC中PCDHGB4表达与免疫检查点途径基因的相关性进行了分析。

### 1.7 PCDHGB4的甲基化分析

UALCAN数据库探索LUSC中的PCDHGB4启动子DNA甲基化水平，以确定肿瘤和正常组织之间的差异，此外还在LUSC样本中评估了PCDHGB4的CpG甲基化状态的预后价值。最后我们使用Spearman相关分析探讨了PCDHGB4与m6A、m5C和m1A调节蛋白之间的相关性。

### 1.8 突变分析

DNA拷贝数数据从cBioPortal数据库下载。在我们的研究中，选择了一共包含1256个样本的LUSC（CPTAC, Cell 2021; TCGA, Firehose Legacy; TCGA, Nature 2012; TCGA, PanCancer Atlas）数据集，分析PCDHGB4的突变情况，并分析了这些突变与患者的临床生存预后之间是否存在关系。

### 1.9 鉴定与PCDHGB4共表达的9个枢纽基因

利用STRING数据库构建PCDHGB4共表达基因的蛋白质-蛋白质相互作用网络。然后，使用GEPIA数据库采用Spearman法确认9个枢纽基因与PCDHGB4之间的表达相关性。此外，应用R包（ggplot2[3.3.6]）对PCDHGB4相关基因进行了基因本体（gene ontology, GO）富集和京都基因和基因组百科全书（Kyoto Encyclopedia of Genes and Genomes, KEGG）通路分析。

### 1.10 功能富集分析

从TCGA数据库LUSC数据集中提取PCDHGB4的数据，并按照PCDHGB4的表达分成高低表达组，利用DESeq2包进行差异分析，按照Padj<0.05、|log2FC|>1的条件筛选差异表达基因。clusterProfiler软件包用于对PCDHGB4差异基因进行富集分析，ggplot2包进行可视化。包括GO富集和KEGG通路分析，GO富集分析包含分子功能、细胞成分和生物学过程三个层面。clusterProfiler包进行GSEA分析，认为|NES|>1、NOM Padj<0.05、FDR Qvalue<0.25是显著富集的。

### 1.11 列线图模型的建立和评估

对TCGA数据库中患者的临床信息进行单因素和多因素Cox回归分析，分析影响 LUSC预后的因素。根据单因素和多因素Cox回归分析的结果，构建列线图模型，校准曲线以评估列线图在1、3和5年的预测精度。

### 1.12 统计学方法

采用各个数据库默认参数进行统计学分析，P<0.05时被视为差异具有统计学意义。

## 2 结果

### 2.1 PCDHGB4在LUSC中的表达及PCDHGB4的亚细胞定位

通过对PCDHGB4 mRNA在33种癌症中的表达情况的分析，我们发现与正常组织相比，PCDHGB4 mRNA在15种肿瘤组织中表达下调，包括膀胱尿路上皮癌、乳腺浸润癌、结肠癌、多形成性胶质细胞瘤、头颈鳞状细胞癌、肾嫌色细胞癌、肾透明细胞癌、肾乳头状细胞癌、肝细胞肝癌、肺腺癌、子宫内膜癌、胰腺癌、前列腺癌、直肠腺癌和LUSC（图1A）。与49例正常组织相比，502例LUSC患者的PCDHGB4 mRNA表达降低（图1B），基因表达综合数据库的107个样本中也得出了同样的结果（图1C）。我们还分析了该基因在NSCLC中的表达情况，如图1D所示，在GSE33532数据集的100个样本中，PCDHGB4 mRNA在NSCLC中低表达。代表性的免疫组化结果见图1E。通过Rh30、SK-MEL-30和U2OS细胞中细胞核、微管和ER的免疫荧光定位获得PCDHGB4亚细胞定位，发现PCDHGB4主要位于核质和质膜中。在SK-MEL-30细胞中，PCDHGB4不仅位于核质和质膜中，还位于细胞溶质中，在Rh30细胞中，PCDHGB4主要位于小囊泡（图1F）。

**图1:**
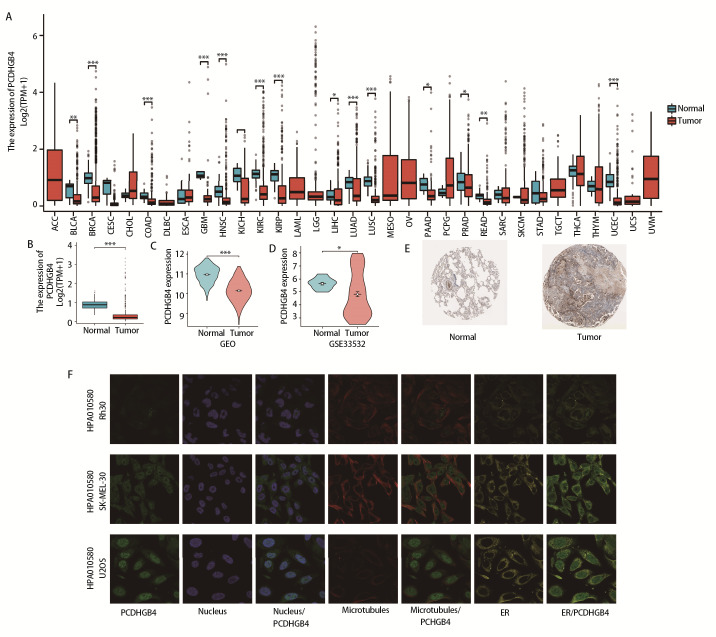
PCDHGB4的表达及定位分析。A：PCDHGB4在泛癌中的表达谱；B、C：正常组织和LUSC组织之间的PCDHGB4基因表达比较；D：正常组织和NSCLC组织之间的PCDHGB4基因表达比较；E：正常组织和LUSC组织中的免疫组织化学图像；F：PCDHGB4亚细胞定位的免疫荧光染色。

### 2.2 PCDHGB4与临床特征的关联

基线资料的比较结果显示，高和低PCDHGB4表达组不同临床特征之间暂未发现明显差异（表1）。然而，箱型图（图2）展示的结果表明PCDHGB4表达与淋巴结转移状态有关，与患者年龄、性别、癌症分期、吸烟习惯或TP53突变之间没有观察到显著相关性。

**表1:** PCDHGB4低表达组和高表达组的LUSC患者的临床病理特征

Characteristics	Low expression of PCDHGB4 (n=205)	High expression of PCDHGB4 (n=188)	X^2^	P
Gender			0.092	0.761
Female	54 (26.3%)	47 (25.0%)		
Male	151 (73.7%)	141 (75.0%)		
Age			0.197	0.657
≤65 yr	83 (40.5%)	72 (38.3%)		
>65 yr	122 (59.5%)	116 (61.7%)		
Smoker			2.216	0.137
No	5 (2.4%)	10 (5.3%)		
Yes	200 (97.6%)	178 (94.7%)		
Pathologic T stage			0.900	0.638
T1	41 (20.0%)	45 (23.9%)		
T2	127 (62.0%)	110 (58.5%)		
T3+T4	37 (18.0%)	33 (17.6%)		
Pathologic N stage			0.255	0.613
N0	128 (62.4%)	122 (64.9%)		
N1+N2+N3	77 (37.6%)	66 (35.1%)		
Pathologic M stage			0.093	0.760
M0	201 (98.0%)	186 (98.9%)		
M1	4 (2.0%)	2 (1.1%)		

Data acquisition: Download and sort out RNAseq data of STAR process of TCGA-LUSC project from TCGA database and extract TPM format data and clinical data; Data filtering strategy: Remove normal+remove no clinical information; Missing value processing: Remove samples with missing variables; Data processing method: log2(value+1); TNM: tumor-node-metastasis; TCGA: The Cancer Genome Atlas.

**图2:**
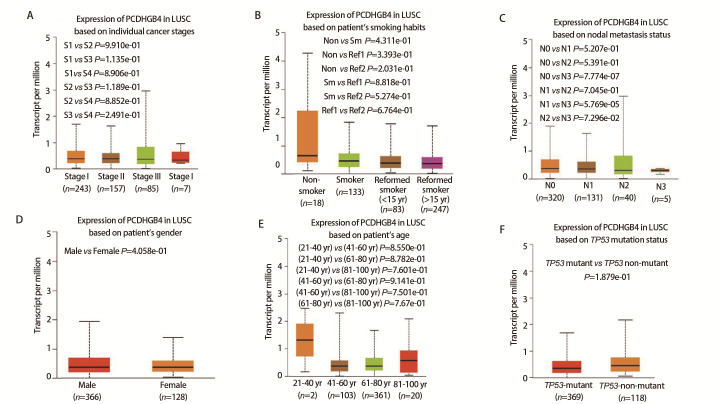
UALCAN数据库中PCDHGB4表达与临床病理特征的相关性。A：肿瘤分期；B：患者吸烟习惯；C：淋巴结转移状态；D：患者性别；E：患者年龄；F：TP53基因突变状态。

### 2.3 PCDHGB4的预后价值

预后分析结果显示PCDHGB4高表达组和低表达组的OS（HR=1.528, P=0.002）、DSS（HR=1.780, P=0.006）和PFS（HR=1.634, P=0.003）均存在差异（图3A-3C），PCDHGB4表达增加与患者较差的预后有关，提示PCDHGB4的表达水平可能对LUSC患者生存预后产生一定影响。同时我们还分析了PCDHGB4的表达水平与NSCLC患者预后之间的关系（图3D-3E），发现PCDHGB4的表达与患者DFS（HR=1.5, P=0.00098）有显著联系。

**图3:**
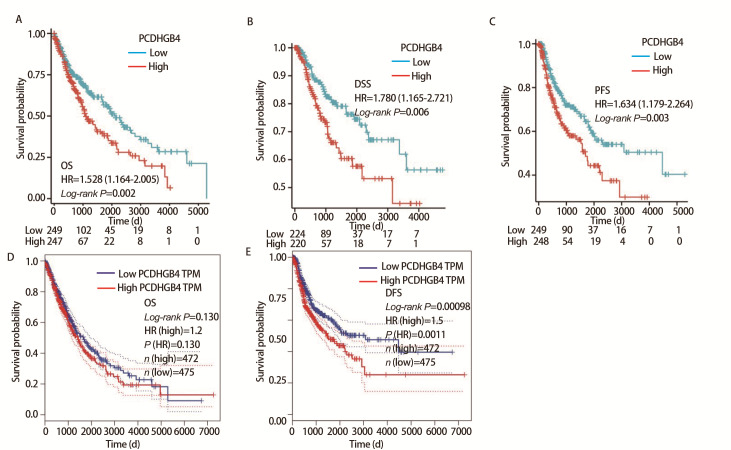
比较PCDHGB4高低表达组之间的预后情况。A-C：PCDHGB4在LUSC中的预后情况（OS、DSS、PFS）；D-E：PCDHGB4在NSCLC中的预后情况（OS、DFS）。

### 2.4 PCDHGB4在癌症中的单细胞分析

我们分析了scTIME网络提供的单细胞测序数据。图4A显示的是40种免疫细胞聚集的可视化图，图4B显示的是PCDHGB4表达在41种免疫细胞中的分布。为了了解在癌症微环境中表达PCDHGB4的主要细胞类型，我们在6个癌症样本的单细胞数据集中对PCDHGB4进行了单细胞分析，图4C中的热图代表了PCDHGB4的表达水平，在包含来自14例NSCLC患者的12,346个细胞的GSE99254数据集中，PCDHGB4主要表达在T细胞和单核细胞或巨噬细胞中（图4D）。在GSE127465数据集中，我们分析了来自7例NSCLC患者的31,179个细胞，发现PCDHGB4在B细胞、T细胞和树突状细胞中表达（图4E）。

**图4:**
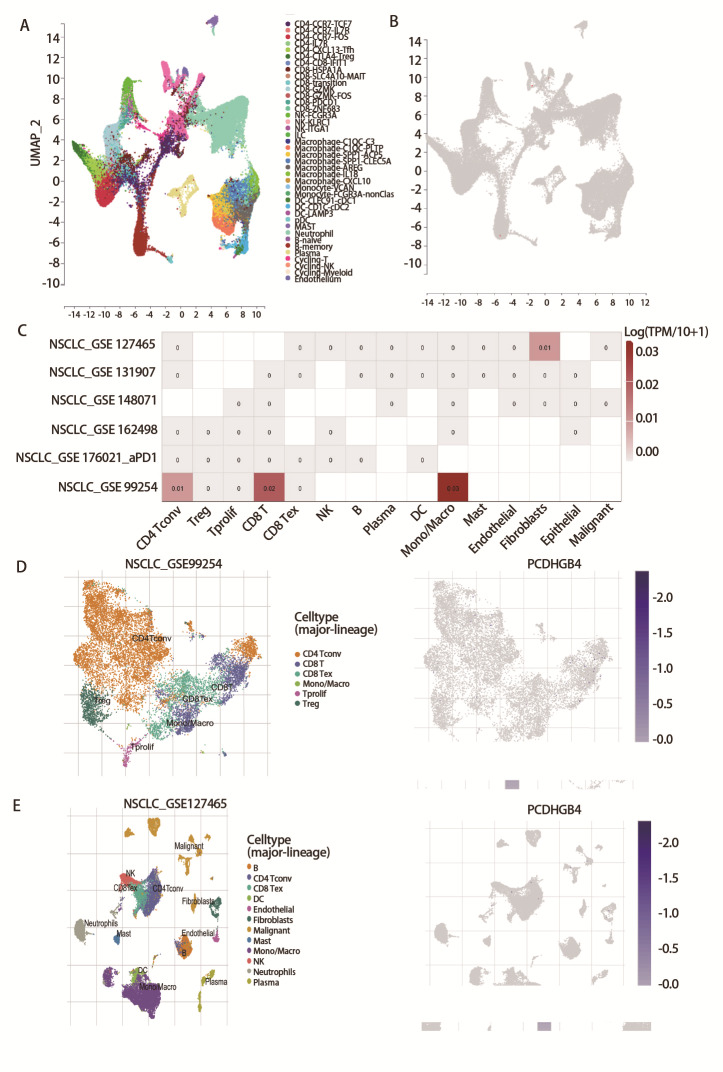
PCDHGB4在LUSC中的单细胞分析。A：PCDHGB4在40种免疫细胞中的聚集情况；B：表现出PCDHGB4表达的免疫细胞；C：6个单细胞数据集中PCDHGB4的表达情况；D：GSE99254 NSCLC数据集中6种不同细胞类型的分布及PCDHGB4的表达水平；E：GSE127465 NSCLC数据集中12种不同细胞类型的分布及PCDHGB4的表达水平。

### 2.5 PCDHGB4与免疫细胞浸润的关系

我们使用Spearman方法探讨了PCDHGB4表达与24种免疫细胞浸润之间的关联。结果显示PCDHGB4与自然杀伤细胞、嗜酸性粒细胞、巨噬细胞、中央记忆型T细胞和效应型记忆T细胞等免疫细胞浸润水平呈正相关，与伽马三角洲T细胞的浸润水平呈负相关（图5A）。选取相关系数绝对值较大的前5位免疫细胞，在LUSC中比较PCDHGB4高表达组和低表达组之间的差别，结果显示在这5种免疫细胞中PCDHGB4高表达组和低表达组之间均存在差异（图5B）。之后，利用ESTIMATE算法计算PCDHGB4与LUSC的免疫浸润评分，包括基质得分（R=0.18, P=5.0e-5）、免疫浸润得分（R=0.11, P=0.01）和估计得分（R=0.15, P=6.5e-4）（图5C）。

**图5:**
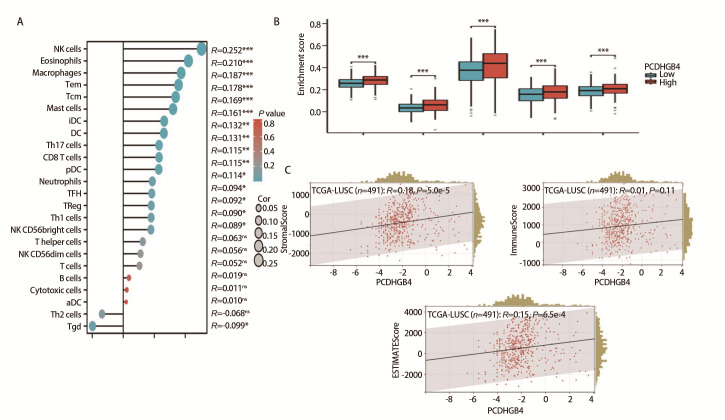
PCDHGB4的表达与免疫细胞浸润有关。A：PCDHGB4和免疫细胞浸润之间的相关性；B：PCDHGB4高、低表达组之间免疫细胞浸润结果的差异；C：PCDHGB4表达与LUSC中基质得分、免疫浸润得分和估计得分的关联。

### 2.6 PCDHGB4与免疫调节基因的关系

我们还研究了PCDHGB4与LUSC中免疫调节基因的关系，包括主要组织相容性复合物、免疫刺激剂，免疫抑制剂、趋化因子和趋化因子受体。结果显示PCDHGB4表达与大多数免疫调节基因显著正相关（图6A、6B）。

**图6:**
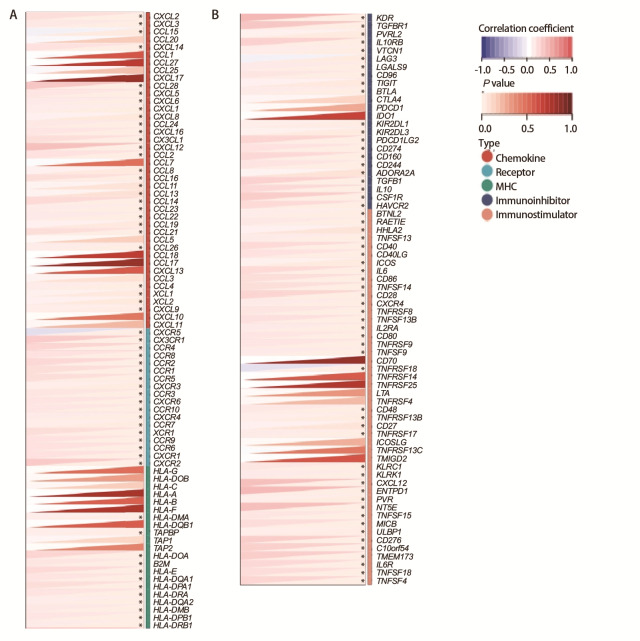
LUSC组织中PCDHGB4与免疫调节基因的相关性分析。A：趋化因子、趋化因子受体和MHC；B：免疫刺激基因和免疫抑制剂基因。

### 2.7 基于免疫检查点基因表达预测肿瘤免疫治疗价值

免疫检查点基因对免疫细胞浸润和免疫治疗有很大影响，我们收集了60个常见的免疫检查点基因，分析了在LUSC中PCDHGB4表达与免疫检查点基因表达的关系，以探索PCDHGB4在免疫治疗中的潜力。结果如图7A所示，在60个免疫检查点基因中有32个与PCDHGB4表达有关，其中与程序性死亡配体1（programmed cell death ligand 1, PD-L1）/CD274、淋巴细胞活化基因3和CD27等呈正相关，与细胞毒性T淋巴细胞相关蛋白4的关系暂未发现。使用TIMER（图7B、7C）和TCGA（图7D、7E）数据库验证了PCDHGB4与常见抑制性检查点分子PD-L1/CD274和刺激性检查点分子CD27的关系，发现在LUSC中PCDHGB4与CD27存在较弱的相关性，与PD-L1的相关性暂未发现。

**图7:**
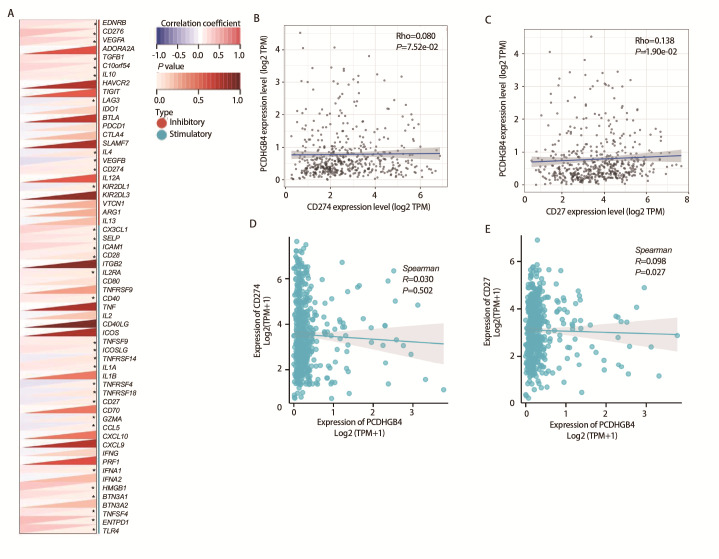
LUSC中PCDHGB4表达与免疫检查点的关系分析。A：PCDHGB4表达与免疫检查点基因表达的相关性分析；B-E：PCDHGB4表达与LUSC中CD274（B、D）和CD27（C、E）的相关性。

### 2.8 PCDHGB4表达与甲基化水平之间的关系

UALCAN数据库检测了PCDHGB4在LUSC中DNA甲基化水平，结果显示LUSC中PCDHGB4的甲基化水平高于正常组织，在不同癌症分期中观察到PCDHGB4甲基化水平存在差异（图8A、8B）。MethSur分析PCDHGB4基因中DNA甲基化水平和CpG岛的预后值。结果显示了7个甲基化的CpG岛，其中cg18509435、cg02758247、cg02050426、cg06823108和cg13161901显示DNA甲基化程度较高（图8C）。此外，2个CpG岛的甲基化水平，即cg06823108和cg13161901与预后有关（P<0.05）（表2）。表明PCDHGB4的甲基化水平有望成为LUSC预后生物标志物。此外，PCDHGB4与大多数癌症中的m6A、m5C和m1A调节基因呈正相关（图8D）。推测PCDHGB4可能通过影响甲基化调节基因的表达水平而产生致癌作用。

**图8:**
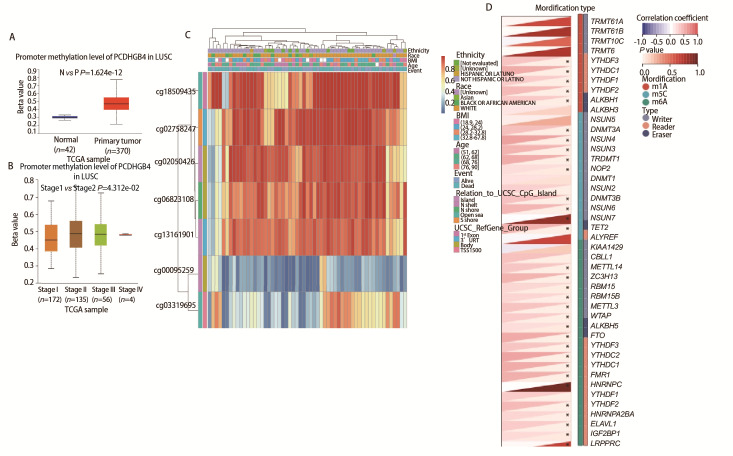
PCDHGB4与甲基化和甲基转移酶的关系。A：LUSC中PCDHGB4启动子甲基化水平；B：在不同肿瘤阶段LUSC组织中PCDHGB4的启动子甲基化水平；C：MethSurv数据库提供的PCDHGB4基因CpG位点DNA甲基化的热图；D：PCDHGB4表达与m1A、m5C、m6A调控基因的相关性。

**表2:** PCDHGB4中CpG的重要预后价值

CpG name	HR (95%CI)	LR test P value	UCSC ref gene group	CpG island (Relation to UCSC)
cg00095259	1.246 (0.907-1.711)	0.177	Body	N_Shelf
cg02050426	0.795 (0.578-1.092)	0.159	Body	Island
cg02758247	0.757 (0.527-1.087)	0.140	3'UTR	S_Shore
cg03319695	1.256 (0.842-1.873)	0.253	TSS1500	Open_Sea
cg06823108	0.653 (0.457-0.933)	0.024	Body	N_Shore
cg13161901	0.700 (0.496-0.988)	0.048	3'UTR	Island
cg18509435	0.818 (0.570-1.175)	0.269	1stExon	Open_Sea
cg25423077	0.723 (0.510-1.025)	0.075	Body	Island

UCSC: University of California Santa Cruz; CI: confidence interval.

### 2.9 PCDHGB4的基因突变与患者的预后无关

我们使用cBioPortal探索PCDHGB4的突变情况，在1.9%的LUSC患者中观察到PCDHGB4基因的突变（图9A）。图9B显示了其他突变及其在PCDHGB4中的位置，“错义”是PCDHGB4的主要突变类型，例如，在3例病例中检测到G361R的改变。通过对PCDHGB4突变与患者的临床生存预后之间关系的研究，我们暂未发现PCDHGB4基因突变与患者的预后生存有关（图9C）。

**图9:**
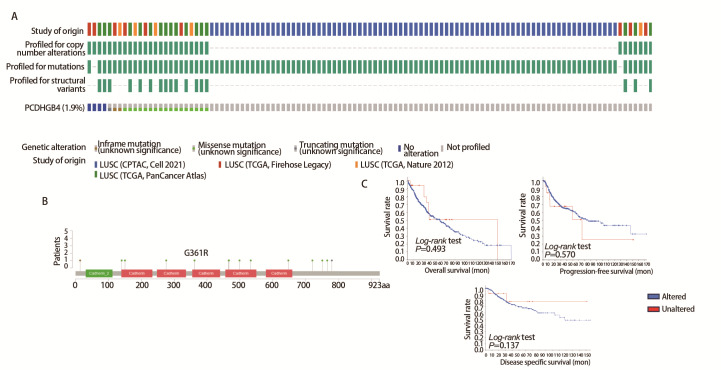
PCDHGB4基因突变与LUSC的生存结果无关。A、B：PCDHGB4不同突变类型的改变频率（A）和突变位点（B）；C：PCDHGB4突变状态与患者预后之间的关系。

### 2.10 鉴定与PCDHGB4共表达的9个枢纽基因表达蛋白

使用STRING，我们鉴定了9个与PCDHGB4共表达的枢纽基因表达蛋白，包括PCDHGB2、PCDHGB1、PCDHGB5、PCDHGB3、CDH19、OR13J1、PTPRD、ASB13和OR6X1（图10A）。接下来，我们使用TCGA数据库检测LUSC中这9个枢纽基因的表达水平。我们的分析表明，PCDHGB3、PCDHGB5、CDH19和PTPRD在LUSC组织中的表达水平降低，而PCDHGB1和ASB13的表达水平升高（图10B）。因此，我们研究了LUSC中9个PCDHGB4相关基因与PCDHGB4之间的表达关联。我们观察到LUSC中PCDHGB4表达与PCDHGB2、PCDHGB1、PCDHGB5、PCDHGB3、CDH19和PTPRD呈正相关（图10C-10K）。我们还对共表达基因进行了KEGG和GO富集分析，以探索LUSC中PCDHGB4相关基因的功能途径。按分子功能、生物过程和KEGG划分的9条富集途径如图10L所示。

**图10:**
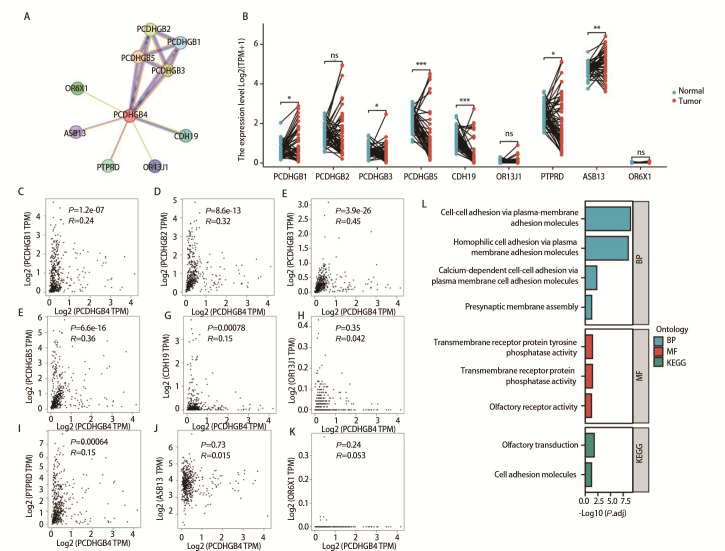
蛋白质相互作用网络与功能富集。A：PCDHGB4相互作用蛋白质网络图；B：9种PCDHGB4相关基因在LUSC的表达；C-K：9种PCDHGB4相关基因的表达与LUSC中PCDHGB4表达的相关性；L：PCDHGB4相关基因的GO和KEGG富集分析。

### 2.11 功能富集

GO和KEGG富集分析表明，在生物学过程中PCDHGB4与质膜黏附分子的细胞间黏附、通过质膜黏附分子的嗜同性细胞黏附和神经视网膜发育有关。分子功能表明PCDHGB4与信号受体激活剂活性和受体配体活性有关。我们发现PCDHGB4相关基因在细胞成分中的片层体和极低密度脂蛋白颗粒处富集。KEGG富集分析显示，PCDHGB4参与了神经活性配体-受体相互作用和PPAR信号通路（图11A）。GSEA富集分析结果显示，PCDHGB4在DNA复制-预启动、RRNA加工和翻译等REACTOME通路显著富集（图11B），在线粒体中的电子传递链氧磷系统、氧化磷酸化和细胞质核糖体蛋白质类等Wiki通路中显著富集（图11C），在核糖体、氧化磷酸化和细胞外基质受体相互作用等KEGG通路显著富集（图11D），在Plateletapp通路、MCM通路和ACE2等Biocarta通路显著富集（图11E）。

**图11:**
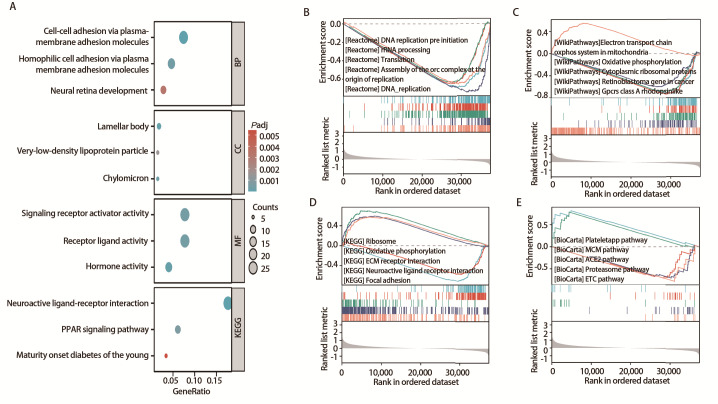
富集分析。A：GO和KEGG富集分析；B-E：GSEA分析。

### 2.12 LUSC患者1、3和5年OS的预后列线图

根据单因素和多因素Cox回归分析的结果（表3），结合PCDHGB4表达和临床病理特征，开发了以T分期、病理分期、年龄和PCDHGB4表达水平为参数的列线图预测模型（图12A），列线图模型的一致性指数为0.609（0.587-0.632）。通过受试者操作特征曲线分析评估了列线图的预测能力。在TCGA-LUSC队列中，1、3和5年OS的列线图曲线下面积分别为0.515、0.629和0.625（图12B）。此外，通过校准曲线来评估列线图的一致性，结果显示1、3和5年OS率校正曲线与理想参考线接近（图12C），表示预测值和实际生存率之间具有良好的一致性。

**表3:** LUSC患者OS的单因素及多因素分析

Characteristics	n	Univariate analysis			Multivariate analysis	
		HR (95%CI)	P		HR (95%CI)	P
Pathologic T stage	496					
T1	114	Reference			Reference	
T2	289	1.237 (0.872-1.753)	0.233		1.135 (0.792-1.626)	0.489
T3+T4	93	1.931 (1.277-2.920)	0.002		1.472 (0.866-2.503)	0.153
Pathologic N stage	490					
N0	317	Reference				
N1	128	1.076 (0.786-1.473)	0.647			
N2+N3	45	1.383 (0.887-2.158)	0.152			
Pathologic stage	492					
I	243	Reference			Reference	
II	159	1.141 (0.829-1.570)	0.419		1.091 (0.775-1.535)	0.619
III+IV	90	1.649 (1.169-2.325)	0.004		1.404 (0.887-2.222)	0.148
Age	490					
≤65 yr	190	Reference			Reference	
>65 yr	300	1.279 (0.960-1.704)	0.093		1.319 (0.987-1.764)	0.061
Gender	496					
Female	130	Reference				
Male	366	1.211 (0.879-1.669)	0.241			
Smoker	484					
No	18	Reference				
Yes	466	0.585 (0.259-1.325)	0.199			
PCDHGB4	496					
Low	249	Reference			Reference	
High	247	1.548 (1.176-2.036)	0.002		1.583 (1.201-2.086)	0.001

Data acquisition: Download and sort out RNAseq data of STAR process of TCGA-LUSC project from TCGA database and extract TPM format data and clinical data. Data filtering strategy: remove normal+remove no clinical information. Missing value processing: Variable missing is not uniformly processed (Pathologic N stage: 6 cases; Pathologic stage: 4 cases; Age: 6 cases; Smoker: 12 cases). Data processing method: log2(value+1).

**图12:**
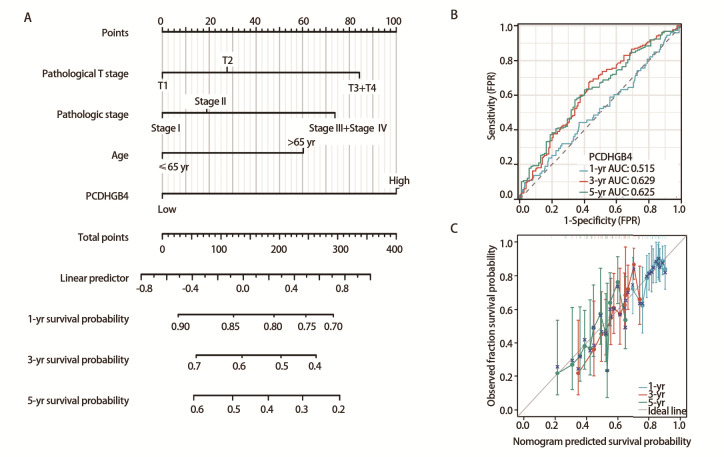
LUSC患者1、3和5年OS的预后列线图。A：列线图模型；B：用于预测TCGA-LUSC队列中1、3和5年OS列线图的ROC曲线；C：用于预测TCGA-LUSC队列中1、3和5年OS的列线图校准曲线。

## 3 讨论

肺癌作为全球癌症死亡的主要原因，一定程度上是由其疾病特征所决定的。早期NSCLC症状和临床表现较轻，发现比较困难，但疾病进展迅速，绝大多数患者直到晚期才被诊断出来，从而导致预后差、死亡率高^[10]^。肿瘤免疫疗法是当前较为火热的癌症治疗方式之一，被誉为癌症治疗的第三次革命。PD-L1表达阳性和高肿瘤突变负荷被认为可以预测NSCLC对免疫治疗的反应，但有研究^[11]^发现一部分LUSC患者只有着很低的受益率，同时还发现超过一半的PD-L1高表达患者对ICIs表现出耐药。可见PD-L1和高肿瘤突变负荷在预测LUSC免疫治疗受益者方面的作用非常有限。因此，开发更有效的生物标志物已成为LUSC免疫疗法的迫切需求^[1]^。钙黏蛋白是细胞表面的一大群跨膜蛋白，包括经典钙黏蛋白、桥粒钙黏蛋白和原钙黏蛋白，参与神经模式、细胞迁移、轴突引导、突触形成和突触功能等过程，同时与大脑进化的多样性也有关^[12,13]^。人类原钙黏蛋白（protocadherin, PCDH）基因位于5号染色体上，主要在大脑中表达，由53个串联排列的基因组成，形成了三个基因簇，包括PCDH-α、PCDH-β和PCDH-γ^[14]^。PCDH家族成员在多种人类恶性肿瘤中异常表达。其中，一项关于钙黏蛋白家族基因成员在乳腺癌中的研究^[15]^显示，大多数家族成员的表达与患者预后、临床病理特征、突变、甲基化以及免疫浸润等多个环节均有不同程度的关系，提示该基因家族成员在癌症发展过程中的重要作用。同时一项对45例NK/T细胞淋巴瘤和33例非NK/T细胞淋巴瘤患者的研究^[16]^发现，PCDH15的表达水平可能有助于非霍奇金淋巴瘤患者的治疗效果判断和预后估计。在肝癌中我们发现PCDH17的表达与患者总体预后以及体内转移相关，PCDH17还可以通过激活表皮生长因子受体/促分裂原活化蛋白激酶/细胞外调节蛋白激酶信号通路来促进肝细胞癌转移^[17]^。PCDH-PC是人类Y染色体上的一个基因，在细胞凋亡和激素抵抗的人前列腺癌细胞中选择性表达，能通过Wnt信号通路诱导前列腺癌细胞神经内分泌转分化^[18]^。PCDH10被确定为一种重要的肿瘤抑制基因，在结直肠癌发生、侵袭和转移中发挥关键作用^[19]^。通过上述研究我们推测PCDH家族成员表达与癌症进展有关，其可能通过抑制癌细胞的增殖和转移来发挥抑制肿瘤的功能^[20]^。PCDHGB4是γ基因簇的成员，属于簇状原钙黏蛋白家族，目前有关PCDHGB4在人类癌症尤其是LUSC中的生物学效应尚未有研究进行详细报道。

我们的研究发现，与正常组织相比，PCDHGB4 mRNA在15种癌症组织中表达下调。与正常肺组织相比，LUSC/NSCLC患者的PCDHGB4 mRNA表达降低。我们还获得了PCDHGB4亚细胞定位，发现PCDHGB4主要位于核质和质膜中，而细胞质中的RNA多在转录后及翻译水平上起调控作用^[21]^，这与我们富集分析得出的结果一致。淋巴结转移作为癌症转移的重要方式之一，在很大程度上影响患者治疗方式的选择与预后，我们的研究发现PCDHGB4表达与淋巴结转移状态有关，曾发现无淋巴结转移的肺癌患者的5年生存率接近75%，有研究^[22]^提示PCDHGB4有望成为判断LUSC预后及淋巴结转移风险的指标。LUSC预后分析显示，PCDHGB4表达增加与患者较差的预后有关，预后列线图模型显示在TCGA-LUSC队列中，1、3和5年预测值和实际生存率之间具有良好的一致性，旨在能够为LUSC患者的生存预测提供简便的工具。基于DNA甲基化水平差异的标志物自发现以来，在肺癌的早期诊断、预测治疗效果和预后评估等方面发挥了重要价值。我们的研究发现LUSC中PCDHGB4甲基化水平高于正常组织，有2个CpG位点的甲基化水平与预后相关，而异常的DNA甲基化通过启动子区域的甲基化沉默了肿瘤抑制基因的表达，作为肿瘤发生中的重要步骤，我们推测DNA甲基化在LUSC的发生和发展中起着重要作用^[23]^。突变分析显示在1.9%的LUSC患者中观察到PCDHGB4基因的突变，“错义”是主要突变类型，但这种基因突变与患者的预后生存暂未在我们的研究中发现。

当前更多的研究关注于基因在免疫微环境中的作用以及对肿瘤进展的影响。单细胞测序分析显示，在肿瘤微环境中PCDHGB4主要在T细胞、单核细胞或巨噬细胞以及树突状细胞等细胞中表达，推测PCDHGB4可能通过塑造肿瘤微环境来影响癌症的发生。我们还发现PCDHGB4表达与多种免疫细胞浸润有关，其中与NK细胞、嗜酸性粒细胞、巨噬细胞、中央记忆型T细胞和效应型记忆T细胞等免疫细胞浸润水平呈正相关，与伽马三角洲T细胞的浸润水平呈负相关，并且这种差异在LUSC中的高和低PCDHGB4表达组之间也存在。既往研究^[24]^表明肺癌的发生与肿瘤微环境关系密切，各种免疫细胞与肺癌患者预后相关，并在抗肿瘤、肿瘤的免疫监视或免疫逃逸、促进肿瘤的发生发展和演进等各方面发挥重要作用。基于ESTIMATE算法计算的免疫浸润评分有助于肿瘤中免疫和基质成分的定量，我们的研究发现PCDHGB4与LUSC组织中免疫浸润得分相关，表明PCDHGB4可能参与LUSC免疫抑制微环境的调控。此外，还发现PCDHGB4表达与大多数免疫调节基因显著正相关。细胞外基质（extracellular matrix, ECM）的异常能使基质细胞的行为失调，从而促进肿瘤相关的血管生成和炎症反应。基于上述发现并结合GSEA富集分析结果，我们推测PCDHGB4表达与LUSC中免疫应答和ECM相关，在LUSC免疫细胞浸润的调控以及肿瘤免疫微环境的重塑中发挥关键作用^[25]^。近年来，由于癌症精准治疗的提出，ICIs便成为了研究热点。对于晚期肺癌患者，免疫疗法成为一种比化疗有效且毒性更小的治疗方法。PD-L1作为迄今为止研究最广泛的ICIs疗法，在NSCLC、黑色素瘤、淋巴瘤和肝细胞癌等疾病中发挥重要作用，其中对转移性NSCLC的生存期有显著的益处，能提高患者中位OS^[26]^。CD27是新一代ICIs，越来越多的证据支持CD27激动剂抗体单独使用或联合PD-L1阻断剂使用，能提高癌症免疫治疗的疗效^[27]^。我们的研究发现，PCDHGB4与大多数免疫检查点基因有关，在LUSC中PCDHGB4与CD27存在较弱的相关性。研究^[28]^发现肿瘤细胞往往通过表达CD27来调节肿瘤微环境中CD70的表达，从而促进免疫逃逸，而高PCDHGB4表达预示着靶向免疫检查点基因免疫疗法的良好治疗效果，据此我们推测PCDHGB4有望作为理想的免疫治疗靶点在LUSC的诊断中发挥作用。

最后，我们鉴定了与PCDHGB4共同表达的枢纽基因，有4种在LUSC组织中表达降低，而PCDHGB1和ASB13的表达水平升高。有研究^[29]^发现PTPRD突变可以作为预测晚期NSCLC接受ICIs治疗的预后生物标志物。最后，我们对差异基因进行了GO、KEGG以及GSEA富集分析。GO分析发现，PCDHGB4参与质膜黏附分子的细胞间黏附，KEGG通路分析表明，PCDHGB4主要与神经活性配体-受体相互作用和PPAR信号通路相关，GSEA分析显示，PCDHGB4主要与氧化磷酸化和ECM受体相互作用有关。有研究^[30]^报道结果表明，由于PPARγ能增加p-EGFR、p-c-MET和Vimentin的表达以及E-cadherin的减少，导致转化生长因子-β诱导PPARγ信号转导促进上皮间充质转化，在肺癌细胞的侵袭和迁移中起至关重要的作用。同时还发现有些信号转导有可能与肺癌药物耐药性有关^[31]^，本研究关于PCDHGB4与PPAR信号通路以及ECM是如何影响LUSC的发生还需要进一步的探究。

综上所述，本研究主要通过生物信息学方法分析PCDHGB4在LUSC中的表达。发现PCDHGB4在LUSC免疫治疗和预后预测方面的潜在价值，并初步探讨了PCDHGB4在LUSC中可能参与的信号通路，这些发现为PCDHGB4在LUSC发生中的作用提供了一定见解。同时我们的研究也存在一定的局限性，本研究的发现大多都基于生物信息学探索，这些回顾性数据可能存在选择偏倚，其结果也有待进一步利用基础和临床实验进行验证，今后采用大样本的前瞻性研究来论证这些发现是很有必要的。
